# Obesity and the Importance of Breathing

**DOI:** 10.7759/cureus.77431

**Published:** 2025-01-14

**Authors:** Bruno Bordoni, Allan R Escher

**Affiliations:** 1 Physical Medicine and Rehabilitation, Foundation Don Carlo Gnocchi, Milan, ITA; 2 Oncologic Sciences, University of South Florida Morsani College of Medicine, Tampa, USA; 3 Anesthesiology/Pain Medicine, H. Lee Moffitt Cancer Center and Research Institute, Tampa, USA

**Keywords:** diaphragm, fascia, obesity, osteopathy, physiotherapy, respiratory rehabilitation

## Abstract

Obesity is a complex and non-communicable disease with a pandemic entity. Currently, multiple causes can lead to obesity, and it is not always easy to create a direct relationship between physical inactivity, poor quality of nutrients consumed, and calculation of excess calories. Among the associated comorbidities, obesity creates a dysfunctional environment of respiratory rhythms at the central and peripheral levels, with functional, morphological, and phenotypic alteration of the diaphragm muscle. This pathological adaptation of breathing is one of the most important causes of the dysregulation of the autonomic system, which will negatively affect the progression of comorbidities and chronic non-physiological adaptations in obese persons. Introducing a physical activity program involving diaphragm training could be a very valid strategy to restore the systemic autonomic response, delaying or avoiding the onset of pathologies in excess fat. This brief narrative review focuses on the importance of breathing in obese subjects.

## Introduction and background

The 2021 Global Burden of Diseases, Injuries, and Risk Factors Study highlights how obesity is progressively increasing after analyzing the risk factors of 204 countries in the world [1]. One in eight people is obese, with a doubling for adults and a quadrupling for minors, compared to the past 30 years; almost three billion people live at the lower limits of obesity (overweight). According to the World Health Organization (WHO), an overweight person has a body mass index (BMI) greater than or equal to 25 kg/m2, while an obese person has a BMI greater than or equal to 30 kg/m2 [2]. Currently, many causes can lead to obesity, and it is not always easy to create a direct relationship between physical inactivity, poor quality of nutrients consumed, and calculating excess calories [3].

WHO defines obesity as: “Obesity is a chronic complex disease defined by excessive fat deposits that can impair health” [4]. Obesity is a complex disease, with a pandemic entity, affecting 37% of the world population [5,6]. Obesity is included in non-communicable diseases, and due to its presence throughout the world, it is called by some authors as “globesity” [7]. From an epidemiological point of view, obesity is more easily found in females, in disadvantaged socioeconomic areas, and in people with low education [7]. In societies with a higher per capita income, obesity affects the population more homogeneously, regardless of sex and age [7]. Forward-looking estimates indicate that in 2030, approximately 78% of American citizens will fall into the obesity/overweight classification, while in Europe, the country most affected by this alteration will be Greece, where in 2035, it is thought that approximately 40% of the population will be obese [7].

Obesity reduces life expectancy by approximately 5-10 years due to the presence of cardiovascular diseases (heart failure, arrhythmias, coronary artery disease, and coronary microvascular disease), type 2 diabetes, and, to a lesser extent, due to the occurrence of tumors [8]. It is a chronic disease and, as such, includes multiple comorbidities: depression, anxiety, dementia, gastrointestinal and genitourinary disorders, muscular and joint problems, skin diseases, respiratory obstructions, and sleep apnea [8]. The constant increase in fat leads to a chronic systemic environment of low-grade inflammation, which lays the foundation for the triggering of the development of major pathologies that increase the mortality and morbidity rate in obese people [9].

An important link connecting obesity and chronic low-grade systemic inflammation is respiratory system dysfunction [9,10]. An alteration of the respiratory system with decreased diaphragm muscle function directly correlates with the levels of inflammation and pro-inflammatory cytokines (interleukins type 1, 6, 8, tumor necrosis factor-α) and C-reactive protein. These inflammatory factors will stimulate the activity of the nuclear factor-κB signaling pathway, which will activate several protein degradation pathways (e.g., the ubiquitin-proteasome system), inducing loss of lean mass (sarcopenia and hypotrophy) [11]. There is an inverse relationship between BMI value and respiratory function inferred from spirometry [12].

This brief narrative review discusses the adaptation of the diaphragm muscle in the presence of obesity. It highlights the importance of respiratory exercise and the usual recommendations for patients with excess fat.

## Review

Obesity and breathing

Obese patients present decreased spirometry values, such as forced vital capacity, forced expiratory volume in 1 second, and peak expiratory flow, in an inverse relationship with respect to BMI values ​​[13]. If body weight increases due to fat mass, respiratory function decreases, with a restrictive pattern [14,15]. In the population, dyspnea is found in a percentage of 10-25%, while in the population of obese individuals, it can reach up to 80% [13]. The diaphragm muscle expresses greater mechanical stress than non-obese and healthy people; the muscle must exert greater contractile force to create intrathoracic pressures during inspiration, with a reduced excursion [13]. Obese patients show a faster but more superficial breathing rhythm compared to healthy subjects [13]. The restrictive pattern resulting from spirometric data does not necessarily indicate diseased lungs; one of the reasons for this restriction can be traced back to the chronic weakness of the diaphragm muscle, which is reflected in a reduced movement of the rib cage [16,17].

In the absence of overt pathologies in obese patients, the lungs do not have restrictive patterns. Still, they expand to a lesser extent due to a reduced capacity for movement of the rib cage, which is linked to a decreased diaphragmatic strength [16]. In fact, a non-optimal expansion of the rib cage (due to the fat, a more positive pleural pressure occurs, becoming a brake on expansion) increases the resistance of the airways, which forces the diaphragm to exert greater contractile force; this mechanism, over time, will cause the diaphragm to weaken [17]. A reduced capacity for thoracic movement creates non-physiological somatic proprioceptive afferents from the muscular and joint area that follow Clarke's column (spinal laminae VII/VIII). This spinal area is a center of convergence between afferents toward the cerebellar area and efferents from the respiratory centers; the entire cerebellar area is involved and influenced by the central pattern generator (CPG), which is located between the pons, midbrain, and medulla [18-21].

A hypermobile thoracic area means altered chronic afferent signals and efferent orders due to the inability to move the rib cage correctly during inspiration. The diaphragm loses a proprioceptive component of the rib cage, decreasing the ability to "understand" how much contractile force to use during respiratory acts.

Leptin resistance

Obesity creates a chronic obesogenic environment. Excessive fat accumulation causes leptin resistance (increased leptin but with poor efficacy). The hormone leptin is part of the central hypothalamic leptin-melanocortin pathway, where leptin stimulates the release of proopiomelanocortin (POMC); POMC will split into ligands, in particular the b isoform (b-melanocyte-stimulating hormone), which in turn will bind and activate the melanocortin-4 receptor. This system is anorectic and allows a greater increase in energy consumption [22]. Leptin resistance, secreted mainly by adipocytes, not only greatly reduces its anorectic and thermogenic effects but also alters the respiratory rate [23].

Leptin can activate some neurons within the nucleus of the solitary tract (part of the CPG); these neurons will directly excite the rostral ventral respiratory area and the dorsomedial area of the hypothalamus, while indirectly, they will excite the area of the neurons that constitute the pre-Bötzinger complex (fundamental for inspiration) [24]. In leptin resistance, the centers of respiratory rhythms and related structures will be disturbed, with an altered expressed function, as in obesity [24].

**Figure 1:**
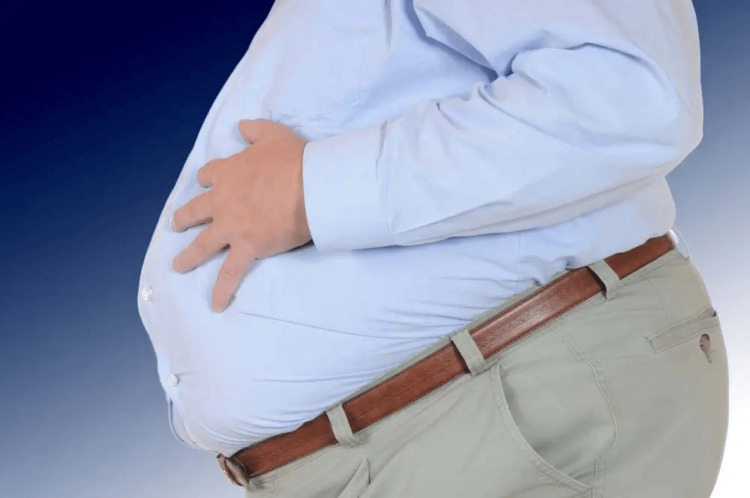
Obesity is included in non-communicable diseases, and due to its presence throughout the world, it is called by some authors as “globesity.” WHO defines a person as overweight if he has a BMI greater than or equal to 25 kg/m2, while a person with obesity has a BMI greater than or equal to 30 kg/m2 WHO: World Health Organization, BMI: body mass index Image Credit: Bordoni Bruno

Obesity creates a dysfunctional environment of respiratory rhythms at the central and peripheral levels (Figure 1) [25].

Obesity and the diaphragm

Adipose cells infiltrate the contractile tissue of the diaphragm muscle, increasing the amount of collagen (type I collagen) and creating a fibrotic environment [26]. In obese people, thrombospondin 1 derived from the extracellular matrix increases its circulating percentage, stimulating the cytokine transforming growth factor β synthesis. The latter leads to the synthesis of adipocytes from muscle mesenchymal cells and greater collagen deposition [27,28]. There are increased oxidation values in the diaphragmatic fibers (with greater protein damage and activity of the cyclooxygenase pathway), for an increase in contractile work required; this stimulates a phenotypic change from white fibers to a greater number of red fibers, but still poorly functional [28].

The organization of the diaphragm fiber loses its functional logic, changing morphology. Alterations such as sarcomere disorganization, thicker Z lines, and non-homogeneous myofibrillar vectors are recorded; vacuoles increase, dysmorphology and mitochondrial dysfunction occur, and there is an increase in lysosomal bodies [28]. The endoplasmic reticulum is dysfunctional, causing a reduction in the creation of contractile force and a cytotoxic environment for calcium deposition [29].

Obese patients show a more cranial diaphragmatic attitude (and decreased functional residual capacity); abdominal and visceral fat pushes the diaphragm cranially [30,31].

We have no data on the presence of obesity and the electrical activity of the phrenic nerve. We have no information on the vagus nerve and the relationship between diaphragm muscle and obesity. The vagus nerve innervates two muscles that constitute the esophageal hiatus, such as the Low's muscle and the transversus intertendinous muscle; these two muscles that derive from the diaphragm are important for a correct relationship during the passage of the bolus. Every time the diaphragm moves, the vagus nerve sends information to the nucleus of the solitary tract (spinosolitary pathway), both for the visceral relationships of the diaphragm and for its relationships with breathing and the parasympathetic system [19].

**Figure 2:**
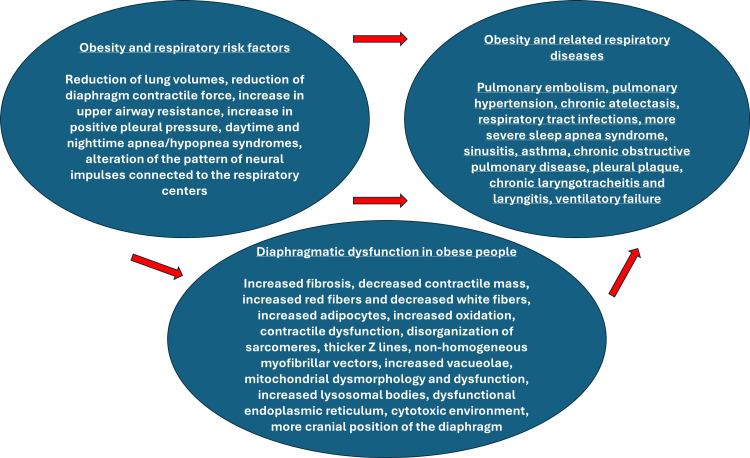
The image summarizes the respiratory risk factors that obesity can cause, the respiratory pathologies that can result from chronic adaptations related to obesity, and the summary of the known adaptations of the diaphragm muscle in the presence of excess fat Image Credit: Bordoni Bruno and Escher Allan

Obesity can increase the risk of many respiratory diseases, reducing lung volumes, reducing the contractile force of the diaphragm, increasing upper airway resistance, increasing pleural pressure, leading to daytime and nighttime apnea/hypopnea syndromes, and altering the pattern of neural impulses connected to the respiratory centers [32]. In the long term, these adaptations will lead to more serious pathologies: pulmonary embolism, pulmonary hypertension, chronic atelectasis, respiratory tract infections, more severe sleep apnea syndrome, sinusitis, asthma, chronic obstructive pulmonary disease, pleural plaque, and chronic laryngotracheitis and laryngitis up to ventilatory failure (Figure 2) [32-34].

Management and prevention of respiratory diseases in obese people

Weight loss is the first challenge that people with obesity must face, but the road to gaining lean mass and losing fat mass is not so simple. Obesogenic memory is linked to chronic epigenetic changes in adipocytes; the body regains the lost weight faster than the time needed to decrease body weight [35]. Another challenge that obese people must undertake is physical activity (aerobic and muscle-strengthening exercises), which must be associated with diet and individualized social and psychological behaviors [36].

Physical activity should include a specific program that helps improve the conditions of the respiratory system, both as prevention and as a possible treatment. Inspiratory muscle training (IMT) seems to improve respiratory muscle performance and the capacity for general body performance. Still, there are controversies about implementing lung function [37]. IMT stimulates the strengthening of the inspiratory muscles through rehabilitation tools, which differ depending on the brand and model. The patient must overcome resistances (pressures) during inhalation with a mouthpiece, which resistances can be changed with a pressure gauge. In this way, the person can perform series and repetitions (number of inhalations), varying the resistance based on the indications of the clinician or therapist. Each series can have the same pressure or be different while maintaining the same number of repetitions. It is the same principle as the athlete who trains in the gym to improve the performance of skeletal muscles; the person manages the weight to be lifted (the pressure during inhalation) with changes in series and repetitions. IMT improves respiratory muscles, including the diaphragm muscle. After IMT, parameters such as maximal inspiratory pressure and voluntary ventilation improve, and endurance capacity and contractile strength increase [38-42]. IMT reduces dyspnea, increases adherence to physical activity, improves general functional capacity, and improves quality of life [43].

IMT is easy to perform, using a low-cost aid that allows for the application of progressive resistances and the following of a variable training program; during the training of a series, the resistance does not change, regardless of the flow rate applied by the patient [44].

Another reason for the recovery of the function of the diaphragm is to improve the parasympathetic system. We know that obesity leads to an increase in the activity of the systemic sympathetic system, although there may be losses of peripheral orthosympathetic endings, for example, from adipose tissue [45,46]. We could hypothesize that the sympathetic system has greater activity due to a decreased capacity for peripheral regulation, or we could suppose that there may be a greater weakness related to the parasympathetic system. The parasympathetic system greatly influences the inflammatory response, controlling the immune response (the inflammatory reflex) [47]. An altered metabolism, as in obesity, generates an inflammatory environment, which is sensed by the surface of immune cells (toll-like receptors) and by some internal portions of each cell (nucleotide-binding oligomerization domain-like receptors) [48,49]. Activating these structures initiates the production of pro-inflammatory cytokines and inflammasomes, which substances are registered by the parasympathetic system, which sends efferents to dampen or eliminate the synthesis of pro-inflammatory cytokines [47]. When the response of the parasympathetic system is deficient or decreases, a chronic metabolic environment of inflammation takes over, as in several chronic pathologies; in obesity, a reduction in the capacity of the parasympathetic system to intervene has been demonstrated [47]. Although there is no real infection, chronic low-grade inflammation due to obesity is present due to a dysregulation of the inflammatory reflex, in which the vagus nerve plays a primary role [50]. The presence of adipocytes with a poorly controlled metabolism (deficiency of sympathetic endings), interconnected with immune cells infiltrated in the adipose tissue, stimulates the production of pro-inflammatory cytokines and adipokines, creating a metabolic endotoxemia (systemic increase of lipopolysaccharide) in the obese person; this condition creates the production of further pro-inflammatory cytokines from other body tissues [50]. The afferents of this condition of dysregulated metabolism and immunological responses are recorded by the peripheral and then spinal endings of the vagus nerve; these afferents will arrive at the nucleus of the solitary tract [50].

In a healthy state, in the presence of inflammation, peripheral efferents of the vagus nerve activate the release of acetylcholine with anti-inflammatory function, which will bind to the alpha7 nicotinic acetylcholine receptor of immune cells to obtain a cholinergic anti-inflammatory response [50]. There is some clinical evidence that stimulation of the vagus nerve (transcutaneous auricular vagus nerve stimulation) can increase the response of anti-inflammatory cells in subjects with metabolic syndrome [51].

Improving the respiratory rhythm, for example, by improving the contractile force of the diaphragm and making the eupneic act deeper, could help the network connected to the nucleus of the solitary tract and the function of the vagus nerve, counteracting the altered immuno-metabolic environment that afflicts the obese patient [52]. Training the diaphragm muscle and reducing upper airway resistance could improve respiratory function and the functions connected to the parasympathetic system, which is dysfunctional in obese patients. Restoring a correct diaphragmatic force can increase the function of the vagus nerve in obese/overweight subjects, including the psychic sphere [53-56].

Stimulating the vagus nerve and the respiratory network by restoring the contractile force of the diaphragm could be an optimal treatment for people with excess fat, helping them lose weight. However, the mechanisms underlying these benefits are still elusive [57]. Furthermore, by improving the mechanics of the thorax and pleura, thanks to a greater capacity for movement of the diaphragm, it is possible to improve the local pulmonary immune response in the presence of viruses and bacteria (preventing their replication) through a correct stimulation of receptors sensitive to mechanical variations (transient receptor potential cation channel subfamily V member 4, receptor for advanced glycation end products) [58]. In obese people, due to altered mechanics of the rib cage, there is difficulty in fighting pulmonary infections [59].

Data are lacking in providing more in-depth answers on the adaptation of obese patients and IMT. Still, preliminary information suggests that including diaphragmatic muscle training may benefit the patient.

To the authors' knowledge, there are no studies in the literature that use manual activities (osteopathy, chiropractic, manual therapy) to improve breathing in obese patients.

What emerges from this brief narrative review is that there are few studies on obese adults and the systemic response following targeted training for the diaphragm muscle, despite the very broad clinical assumptions. Recent works highlight the positive effects on obese children and adolescents and IMT, such as the reduction of dyspnea, the increase in inspiratory force, and the increase in active physical activity performed [60,61]. Obese patients with concomitant respiratory pathologies demonstrate, through IMT, an increase in the thickness of the diaphragm [62]. To obtain further benefits, IMT could include training based on some classifications that group obese patients into subcategories: metabolically healthy obese, metabolically abnormal obese, metabolically obese normal weight, sarcopenic obesity, or a classification according to the severity of the BMI, which are class I (from a minimum of 30 to a maximum of 35 kg/m2), class II (from a minimum of 35 to a maximum of 40 kg/m2), and class III (from 40 kg/m2 and above) [63,64]. We do not have sufficient data on how the diaphragm and breathing adapt according to the sub-classifications of obese people [63]. Furthermore, these groupings are not always univocal in the literature [65,66].

Training improves the proprioception of the rib cage and diaphragm, improving the expressed strength and function. Since obesity alters the function of the respiratory centers, an IMT restores the afferents that derive from the diaphragm to the respiratory centers, improving the neuro-coordination of the respiratory centers toward the periphery. IMT stimulates the position of the diaphragm toward the caudality, contrasting the expiration position of obese people (cranial position). Research carried out with IMT does not have significant follow-up, stating that IMT does not affect lung volumes, which contrasts with the improvement of the diaphragm function. Only one recent study, a follow-up of one year, highlights the improvement of lung parameters in obese children and adolescents (forced expiratory volume in the first 1 second, forced vital capacity) [67].

Research must make a further effort to evaluate the therapeutic association of IMT and the psycho-physical well-being of people with excess fat.

## Conclusions

The 2021 Global Burden of Diseases, Injuries, and Risk Factors Study highlights the significant pandemic of obesity, with approximately one billion people suffering from excess weight. Currently, multiple causes can lead to obesity, and it is not always easy to create a direct relationship between physical inactivity, poor quality of nutrients consumed, and calculation of excess calories. An important link that connects obesity and chronic low-grade systemic inflammation is the dysfunction of the respiratory system. The diaphragm muscle in obese people suffers from functional decline and morphological and phenotypic alteration. Physical activity should include a specific path that helps improve the conditions of the respiratory system, both as prevention and as a possible treatment. IMT seems to promote an improvement in the performance of the respiratory muscles and the body's performance capacity in general. Currently, we have no gold standard indications for IMT in obese subjects, a gap that should be filled for the beneficial clinical prerequisites.

## References

[1] (2024). Global burden and strength of evidence for 88 risk factors in 204 countries and 811 subnational locations, 1990-2021: a systematic analysis for the Global Burden of Disease Study 2021. Lancet.

[2] (2025). Global, regional, and national progress towards the 2030 global nutrition targets and forecasts to 2050: a systematic analysis for the Global Burden of Disease Study 2021. Lancet.

[3] (2020). Global burden of 87 risk factors in 204 countries and territories, 1990-2019: a systematic analysis for the Global Burden of Disease Study 2019. Lancet.

[4] (2024). Obesity and Overweight. https://www.who.int/news-room/fact-sheets/detail/obesity-and-overweight.

[5] Anderer S (2024). One in 8 people worldwide are obese. JAMA.

[6] Islam AN, Sultana H, Nazmul Hassan Refat M, Farhana Z, Abdulbasah Kamil A, Meshbahur Rahman M (2024). The global burden of overweight-obesity and its association with economic status, benefiting from STEPs survey of WHO member states: a meta-analysis. Prev Med Rep.

[7] Koliaki C, Dalamaga M, Liatis S (2023). Update on the obesity epidemic: after the sudden rise, is the upward trajectory beginning to flatten?. Curr Obes Rep.

[8] Fruh SM (2017). Obesity: risk factors, complications, and strategies for sustainable long-term weight management. J Am Assoc Nurse Pract.

[9] Cifuentes M, Verdejo HE, Castro PF (2025). Low-grade chronic inflammation: a shared mechanism for chronic diseases. Physiology (Bethesda).

[10] Costanzo S, Magnacca S, Bonaccio M (2021). Reduced pulmonary function, low-grade inflammation and increased risk of total and cardiovascular mortality in a general adult population: Prospective results from the Moli-sani study. Respir Med.

[11] Cao Y, Li P, Wang Y, Liu X, Wu W (2022). Diaphragm dysfunction and rehabilitation strategy in patients with chronic obstructive pulmonary disease. Front Physiol.

[12] Cesanelli L, Cesanelli F, Degens H, Satkunskiene D (2024). Obesity-related reduced spirometry and altered breathing pattern are associated with mechanical disadvantage of the diaphragm. Respir Physiol Neurobiol.

[13] Alqarni AA, Aldhahir AM, Alqahtani JS (2024). Spirometry profiles of overweight and obese individuals with unexplained dyspnea in Saudi Arabia. Heliyon.

[14] Wang S, Sun X, Hsia TC, Lin X, Li M (2017). The effects of body mass index on spirometry tests among adults in Xi'an, China. Medicine (Baltimore).

[15] Melo LC, Silva MA, Calles AC (2014). Obesity and lung function: a systematic review. Einstein (Sao Paulo).

[16] Lo Mauro A, Tringali G, Codecasa F, Abbruzzese L, Sartorio A, Aliverti A (2023). Pulmonary and chest wall function in obese adults. Sci Rep.

[17] Bordoni B, Kotha R, Escher AR (2024). Symptoms arising from the diaphragm muscle: function and dysfunction. Cureus.

[18] Cho M (2010). Spinal convergence of motor and sensory pathways. Nat Neurosci.

[19] Bordoni B, Escher AR (2021). Functional evaluation of the diaphragm with a noninvasive test. J Osteopath Med.

[20] Bordoni B, Escher A, Compalati E, Mapelli L, Toccafondi A (2023). The importance of the diaphragm in neuromotor function in the patient with chronic obstructive pulmonary disease. Int J Chron Obstruct Pulmon Dis.

[21] Bordoni B, Mapelli L, Toccafondi A (2024). Post-myocardial infarction rehabilitation: the absence in the rehabilitation process of the diaphragm muscle. Int J Gen Med.

[22] Sridhar GR, Gumpeny L (2024). Melanocortin 4 receptor mutation in obesity. World J Exp Med.

[23] Obradovic M, Sudar-Milovanovic E, Soskic S (2021). Leptin and obesity: role and clinical implication. Front Endocrinol (Lausanne).

[24] Krohn F, Novello M, van der Giessen RS, De Zeeuw CI, Pel JJ, Bosman LW (2023). The integrated brain network that controls respiration. Elife.

[25] Chlif M, Keochkerian D, Choquet D, Vaidie A, Ahmaidi S (2009). Effects of obesity on breathing pattern, ventilatory neural drive and mechanics. Respir Physiol Neurobiol.

[26] Buras ED, Converso-Baran K, Davis CS (2019). Fibro-adipogenic remodeling of the diaphragm in obesity-associated respiratory dysfunction. Diabetes.

[27] Buras ED, Woo MS, Kaul Verma R (2024). Thrombospondin-1 promotes fibro-adipogenic stromal expansion and contractile dysfunction of the diaphragm in obesity. JCI Insight.

[28] Rodrigues GC, Rocha NN, Maia LA (2020). Impact of experimental obesity on diaphragm structure, function, and bioenergetics. J Appl Physiol (1985).

[29] Xiang X, Zhu Y, Pan X (2023). ER stress aggravates diaphragm weakness through activating PERK/JNK signaling in obesity hypoventilation syndrome. Obesity (Silver Spring).

[30] Zerah F, Harf A, Perlemuter L, Lorino H, Lorino AM, Atlan G (1993). Effects of obesity on respiratory resistance. Chest.

[31] Boriek AM, Lopez MA, Velasco C (2017). Obesity modulates diaphragm curvature in subjects with and without COPD. Am J Physiol Regul Integr Comp Physiol.

[32] Shah NM, Kaltsakas G (2023). Respiratory complications of obesity: from early changes to respiratory failure. Breathe (Sheff).

[33] Min J, Feng R, Badesch D (2020). Obesity in pulmonary arterial hypertension (PAH): the Pulmonary Hypertension Association Registry (Phar). Ann Am Thorac Soc.

[34] Yang W, Yang Y, Guo Y, Guo J, Ma M, Han B (2023). Obesity and risk for respiratory diseases: a Mendelian randomization study. Front Endocrinol (Lausanne).

[35] Hinte LC, Castellano-Castillo D, Ghosh A (2024). Adipose tissue retains an epigenetic memory of obesity after weight loss. Nature.

[36] Raiman L, Amarnani R, Abdur-Rahman M, Marshall A, Mani-Babu S (2023). The role of physical activity in obesity: let's actively manage obesity. Clin Med (Lond).

[37] Caicedo-Trujillo S, Torres-Castro R, Vasconcello-Castillo L (2023). Inspiratory muscle training in patients with obesity: a systematic review and meta-analysis. Front Med (Lausanne).

[38] Tenório LH, Santos AC, Câmara Neto JB, Amaral FJ, Passos VM, Lima AM, Brasileiro-Santos Mdo S (2013). The influence of inspiratory muscle training on diaphragmatic mobility, pulmonary function and maximum respiratory pressures in morbidly obese individuals: a pilot study. Disabil Rehabil.

[39] Barbalho-Moulim MC, Miguel GP, Forti EM, Campos Fdo A, Costa D (2011). Effects of preoperative inspiratory muscle training in obese women undergoing open bariatric surgery: respiratory muscle strength, lung volumes, and diaphragmatic excursion. Clinics (Sao Paulo).

[40] Edwards AM, Maguire GP, Graham D, Boland V, Richardson G (2012). Four weeks of inspiratory muscle training improves self-paced walking performance in overweight and obese adults: a randomised controlled trial. J Obes.

[41] Casali CC, Pereira AP, Martinez JA, de Souza HC, Gastaldi AC (2011). Effects of inspiratory muscle training on muscular and pulmonary function after bariatric surgery in obese patients. Obes Surg.

[42] Edwards AM, Graham D, Bloxham S, Maguire GP (2016). Efficacy of inspiratory muscle training as a practical and minimally intrusive technique to aid functional fitness among adults with obesity. Respir Physiol Neurobiol.

[43] Villiot-Danger JC, Villiot-Danger E, Borel JC, Pépin JL, Wuyam B, Vergès S (2011). Respiratory muscle endurance training in obese patients. Int J Obes (Lond).

[44] Geddes EL, Reid WD, Crowe J, O'Brien K, Brooks D (2005). Inspiratory muscle training in adults with chronic obstructive pulmonary disease: a systematic review. Respir Med.

[45] Sakamoto K, Butera MA, Zhou C (2025). Overnutrition causes insulin resistance and metabolic disorder through increased sympathetic nervous system activity. Cell Metab.

[46] Leon-Mercado L, Gautron L (2025). Sympathetic NPY boosts thermogenic adipocytes. Am J Physiol Endocrinol Metab.

[47] Pavlov VA, Tracey KJ (2012). The vagus nerve and the inflammatory reflex--linking immunity and metabolism. Nat Rev Endocrinol.

[48] Apaza CJ, Días M, García Tejedor A, Boscá L, Laparra Llopis JM (2024). Contribution of nucleotide-binding oligomerization domain-like (NOD) receptors to the immune and metabolic health. Biomedicines.

[49] Sameer AS, Nissar S (2021). Toll-like receptors (TLRs): structure, functions, signaling, and role of their polymorphisms in colorectal cancer susceptibility. Biomed Res Int.

[50] Pavlov VA (2021). The evolving obesity challenge: targeting the vagus nerve and the inflammatory reflex in the response. Pharmacol Ther.

[51] de Moraes TL, Costa FO, Cabral DG (2023). Brief periods of transcutaneous auricular vagus nerve stimulation improve autonomic balance and alter circulating monocytes and endothelial cells in patients with metabolic syndrome: a pilot study. Bioelectron Med.

[52] Szulczewski MT (2022). Transcutaneous auricular vagus nerve stimulation combined with slow breathing: speculations on potential applications and technical considerations. Neuromodulation.

[53] Sato K, Kawamura T, Yamagiwa S (2010). The "Senobi" breathing exercise is recommended as first line treatment for obesity. Biomed Res.

[54] Sato K, Kawamura T, Yamagiwa S (2011). The "Senobi" breathing exercise ameliorates depression in obese women through up-regulation of sympathetic nerve activity and hormone secretion. Biomed Res.

[55] Sato K, Kawamura T, Abo T (2010). "Senobi" stretch ameliorates asthma symptoms by restoring autonomic nervous system balance. J Investig Med.

[56] Sant' Anna M Jr, Carvalhal RF, Carneiro JR, Lapa MS, Zin WA, Lugon JR, Guimarães FS (2014). Association between respiratory mechanics and autonomic function in morbid obesity. Rev Port Pneumol.

[57] de Lartigue G (2016). Role of the vagus nerve in the development and treatment of diet-induced obesity. J Physiol.

[58] Bai H, Si L, Jiang A (2022). Mechanical control of innate immune responses against viral infection revealed in a human lung alveolus chip. Nat Commun.

[59] Almond M, Farne HA, Jackson MM (2023). Obesity dysregulates the pulmonary antiviral immune response. Nat Commun.

[60] Lang JE, Carrion VM, Bhammar DM, Howard JB, Armstrong SC (2024). A randomized trial of inspiratory training in children and adolescents with obesity. Child Obes.

[61] Kaeotawee P, Udomittipong K, Nimmannit A, Tovichien P, Palamit A, Charoensitisup P, Mahoran K (2022). Effect of threshold inspiratory muscle training on functional fitness and respiratory muscle strength compared to incentive spirometry in children and adolescents with obesity: a randomized controlled trial. Front Pediatr.

[62] Cheng YY, Lin SY, Hsu CY, Fu PK (2022). Respiratory muscle training can improve cognition, lung function, and diaphragmatic thickness fraction in male and non-obese patients with chronic obstructive pulmonary disease: a prospective study. J Pers Med.

[63] Mayoral LP, Andrade GM, Mayoral EP (2020). Obesity subtypes, related biomarkers &amp; heterogeneity. Indian J Med Res.

[64] Sun JY, Huang WJ, Hua Y (2022). Trends in general and abdominal obesity in US adults: Evidence from the National Health and Nutrition Examination Survey (2001-2018). Front Public Health.

[65] De Lorenzo A, Soldati L, Sarlo F, Calvani M, Di Lorenzo N, Di Renzo L (2016). New obesity classification criteria as a tool for bariatric surgery indication. World J Gastroenterol.

[66] Lin Z, Feng W, Liu Y (2021). Machine learning to identify metabolic subtypes of obesity: a multi-center study. Front Endocrinol (Lausanne).

[67] Charoensittisup P, Udomittipong K, Mahoran K, Palamit A (2024). Longitudinal effects of obesity on pulmonary function in obese children and adolescents. Pediatr Res.

